# Validation Study of the Estimated Glycemic Load Model Using Commercially Available Fast Foods

**DOI:** 10.3389/fnut.2022.892403

**Published:** 2022-05-10

**Authors:** Miran Lee, Haejin Kang, Sang-Jin Chung, Kisun Nam, Yoo Kyoung Park

**Affiliations:** ^1^Department of Medical Nutrition, Graduate School of East-West Medical Science, Kyung Hee University, Yongin, South Korea; ^2^Department of Medical Nutrition (AgeTech-Service Convergence Major), Kyung Hee University, Yongin, South Korea; ^3^Department of Foods and Nutrition, Kookmin University, Seoul, South Korea; ^4^Pulmuone Co., Ltd., Seoul, South Korea; ^5^Department of Food Innovation and Health, Graduate School of East-West Medical Science, Kyung Hee University, Yongin, South Korea

**Keywords:** glycemic index (GI), glycemic load (GL), diet, carbohydrate loading, fast foods

## Abstract

The recent popularization of low-glycemic foods has expanded interest in glycemic index (GI) not only among diabetic patients but also healthy people. The purpose of this study is to validate the estimated glycemic load model (eGL) developed in 2018. This study measured the glycemic load (GL) of 24 fast foods in the market in 20 subjects. Then, the transportability of the model was assessed, followed by an assessment of model calibration and discrimination based on model performance. The transportability assessment showed that the subjects at the time of model development are different from the subjects of this validation study. Therefore, the model can be described as transportable. As for the model's performance, the calibration assessment found an *x*^2^ value of 11.607 and a *p*-value of 0.160, which indicates that the prediction model fits the observations. The discrimination assessment found a discrimination accuracy exceeding 0.5 (57.1%), which confirms that the performance and stability of the prediction model can be discriminated across all classifications. The correlation coefficient between GLs and eGLs measured from the 24 fast foods was statistically significant at 0.712 (*p* < 0.01), indicating a strong positive linear relationship. The explanatory powers of GL and eGL was high at 50.7%. The findings of this study suggest that this prediction model will greatly contribute to healthy food choices because it allows for predicting blood glucose responses solely based on the nutrient content labeled on the fast foods.

## Introduction

It has been reported that excessive carbohydrate intake causes obesity and diabetes, which lead to claims that carbohydrate intake should be controlled (1). These trends also expanded interest and research in the low-carbohydrate diet, where carbohydrates account for 45% or lower of the total energy intake, or ultra low-carbohydrate diet, where the percentage is 10% or lower (2). It has been also suggested that it is positive to choose foods with low glycemic index (GI), thereby slowing down digestion and absorption and controlling appetite in the short term, and interest in low-GI foods continues today (3). GI reflects the digestion and absorption speed of carbohydrates in a food. GI is measured by comparing the blood glucose response of a food after consumption with the blood glucose response of the reference foods, and expressed in percentage (4). Atkinson et al. (5) classified a food with a GI of 55 or lower as a low-GI food, a food with a GI above 55 and below 70 as a medium-GI food, and those with a GI exceeding 70 as a high-GI food. It has been reported that low-GI diet slows down carbohydrate digestion and absorption, increases satiety, and improves blood lipids and insulin resistance, thereby mitigating risks of cardiovascular diseases, diabetes, and obesity (6). The Korean Food Sanitation Act still does not allow GI to be included in processed food labels, whereas in Australia, for example, the Glycemic Index Foundation (GIF) allows for GI labeling on food packaging through the “Low GI Symbol Program” (7).

The recent popularization of low-glycemic foods has expanded interest in GI not only among diabetic patients but also healthy people (8). However, GI does not take single-time carbohydrates intake into account. To address this shortcoming, we need to consider glycemic load (GL) to quantify the glycemic effect included in a single portion of a food (9). A food with a GL of 10 or lower has been classified as a low-GL food, whereas a food with a GL over 10 and below 20 has been classified as a medium-GL food, and a food with a GL of 20 or higher has been classified as a high GL food (10). The type of carbohydrate (potato, bread, rice, etc.) and the food consumed with carbohydrates affects glycemic indicators such as GI and GL (11). Lee et al. (12) developed the estimated glycemic load model (eGL) equation for Koreans, who mostly rely on mixed diets rather than consuming a single food containing carbohydrate, to address the complexity and inaccuracy of glycemic calculation for mixed diets. Subsequently, the diets of Korean adults were evaluated using the data from the Korea National Health and Nutrition Examination Survey (KNHANES) and the developed eGL prediction model, to verify the usefulness of the model (13). As such, this study aims to review the validity of the eGL prediction model calculated using a number of mixed meal replacements.

## Materials and Methods

### Research Design

The survey took from July 15 to September 21, 2019 at Kyung Hee University and Kookmin University under the approval of the Institutional Review Board of Kyung Hee University Global Campus (approval number: KHGIRB-19-147, approval date: June 26, 2019). Subjects were recruited through an open call process and were briefed about the research process during the first visit. Then, the research continued with the subjects who, after reading the research subject manual, voluntarily agreed to participate in the research and signed the research subject consent form. The blood glucose levels of the subjects were measured at 0 min before food consumption, and 15, 30, 45, 60, 90, and 120 min after consumption. All subjects were provided with test food between 7 and 11 a.m., and the intake was completed within 15 min. The measurements were recorded in a blood glucose measurement record sheet. The subjects were arbitrarily divided into two groups, and were visited 13–14 times throughout the research period to measure blood glucose levels.

### Subjects

The subjects were selected from healthy adults with normal fasting blood glucose levels and no significant health-related issues, aged between 20 and 45 years. They were recruited by posting a call for subjects indicating the selection and exclusion criteria at the universities. The exclusion criteria included: history of diabetes in immediate family members; chronic diseases or endocrine diseases such as thyroid diseases; pathophysiological risk factors such as digestive disorders; inability to go through self-blood glucose test using a blood glucose tester due to psychological fear; body mass index (BMI) of 25 kg/m^2^ or higher (obesity) for Asian defined by World Health Organization/International Association for the Study of Obesity/International Obesity Task Force (WHO/IASO/IOTF) (14). An oral glucose tolerance test (OGTT) was conducted during the first visit, to screen out subjects with a fasting blood glucose level of 100 mg/dL or higher and those with a blood glucose level of 140 mg/dL or higher 2 h after consuming glucose solution. In addition, subjects were excluded if they failed to complete four baseline blood glucose response tests (two with glucose solution, two with steamed white rice) or could not continue participation due to health issues. In accordance with the International Organization for Standardization (ISO) standards (15), blood glucose tests were conducted only on subjects who did not consume alcohol on the previous day, maintained normal diet and sleep, and did not engage in intense exercises on the morning of the visitation day. The subjects were informed of this before each test. In addition, tests were conducted after confirming whether the subjects did not consume any food including water for at least 10 h. ISO technical committees which suggest 10 subjects per food item is recommended for measuring GI.

### Research Method

#### Body Composition Analysis

During the first visit, the body components of the subjects on empty stomach were measured using a body fat analyzer (InBody 270, InBody Co., Ltd.).

#### Baseline Blood Glucose Response Measurements

The ISO standards clearly specify the standard method for determining the GI of a food. Reference foods were selected based on the ISO standards, and the blood glucose curve response of each subject was measured in advance. A reference food is defined as a food with around 100 GI. In this study, all subjects were instructed to consume the reference foods, and their individual blood glucose response curves were measured. Glucose, white bread, and rice have been proposed as reference foods because they have more standardized carbohydrate content than other foods, and show fewer fluctuations in GI values (15, 16). In addition, the blood glucose response of a reference food is expressed as the incremental area under the blood glucose response curve (IAUC). It is recommended to conduct at least In review two blood glucose response tests on a separate day during the research period. In this study, the following foods were selected as reference foods allowed under the above standards: glucose solution (dextrose, anhydrous, 50 g); and steamed white rice (154 g, glucose content around 50 g), which is a carbohydrate food enjoyed by most Koreans. Then, two tests were conducted for each in accordance with the ISO standards.

#### Food Consumption and Blood Glucose Measurements

After the baseline blood glucose response tests, subjects consumed the reference foods four times and different processed foods nine to ten times. Each subject was visited 13–14 times. Each food was consumed by eight different subjects, followed by blood glucose measurements. Subjects measured their own blood glucose levels using ACCU-CHEK Performa (Roche Diagnostics Korea Co., Ltd) at 0 min before food intake and 15, 30, 45, 60, 90, and 120 min after food intake. Each subject took his/her blood glucose measurements by washing the hands before the test, completely removing moisture, disinfecting the area with alcohol wipes, and then taking blood from the tip of a finger using the needle of the tester. The subjects were instructed to record their first blood glucose measurements. During the test, the subjects were instructed to refrain from water consumption. If a subject wanted, he/she was allowed to consume 100 mL or less of water. To rule out the effect of blood glucose response, the subjects were instructed to sit and refrain from speaking or making big movements during the 2-h measurement.

### Test Food Selection

In order to validate the estimated eGL prediction model developed in a previous study, this study selected 24 prediction models in the market that contain carbohydrate (g), protein (g), fat (g), and dietary fiber (g) based on the nutrient labels. The 24 processed foods were selected to vary the carbohydrate, processed food, fat, and dietary fiber contents. The selected foods included: two types of bread (bulgogi croquette and sponge cake); three types of calorie control foods (balance shake, sweet potato health meal cold/hot, and tofu and lentil rice meal); two types of cereals (cereal and whole-grain cereal); two types of In review dumplings [dumplings with kimchi (frozen) and dumplings with meat (frozen)]; three types of readymade rice [bibimbap (frozen), fried rice with hamburger steak (frozen), and fried rice with shrimp (frozen)]; a type of hot dog (cheese and sausage hot dog); three types of noodles (cream past, spicy noodle, and tomato pasta); two types of porridge (beef and mushroom rice porridge and red bean porridge); a type of salad (corn salad); two types of snacks (dried tofu snack and almond cookies); a type of soup (button mushroom soup); and a type of tteokbokki (wheat noodle tteokbokki). Each food was distributed between the two subject groups. All foods were served after preparing them with microwave ovens in accordance with the instructions indicated on the food packaging. **Table 4** lists the 24 processed foods energy and nutrient values.

### GI, GL, and eGL Equations

The blood glucose measurements were analyzed using Graphpad Prism 8.3.0 to calculate their IAUCs. Then, GI (17) and GL (5) were calculated using the IAUC values and the following equations.


$$\begin{array}{l} GI\;=\;IAUC\;after\;consuming\;the\;processed\;foods\\ \;÷\;IAUC\;after\;consuming\;glucose\;solution\;\times \;100\\ GL\;=\;GI\;\times \;available\;carbohydrate(carbohydrate\\ \;-\;dietary\;fiber)\;÷\;100 \end{array}$$

The carbohydrate, dietary fiber, prediction model, fat, and other nutrient content in **Table 3** were applied to the eGL prediction model equation (13) to calculate eGLs.


$$\begin{array}{l} eGL\;=\;a\;+\;[b\;\times \text{ (}carbohydrate\;-\;dietary\;fiber\text{)}]\;-\;(c\;\times \;fat)\\ \;-\;(d\;\times \;protein\;\times \;protein)\;-\;(e\;\times \;dietary\;fiber\\ \;\times \;dietary\;fiber) \end{array}$$

### Statistical Analysis

The validation of a prediction model hinges on how well the model works for the subjects who did not participate at the time of the model's development (18). To validate the model, the transportability of the model was assessed, followed by an assessment of model calibration and discrimination based on model performance. Model transportability assessment is a method to verify whether the validity of the model can be ensured with different subjects, research period, and research organizations. It has been reported that transportability can be only assessed in external validation but is not always included in the assessment (19, 20). After the transportability assessment, the performance of the model was assessed to understand how suitable the prediction model is for application to new subjects (21). First, through the transportability assessment, the kai square test and the independent sample *T*-test were used to verify the general characteristics of the two group and the difference between the two groups in terms of the factors included in the eGL prediction model. In addition, the logistic regression analysis was used to calculate the regression coefficients and standard errors of the factors included in the model and verify their calibration. The calibration assessment verifies how closely the values predicted using a prediction model match the observed values. The discrimination assessment indicates how the observed values and the predicted values are discriminated in sub-groups, not the overall subjects. In the calibration assessment, the subjects were classified based on their genders, BMIs, and body fat for the Hosmer-Lemeshow goodness-of-fit test. Lastly, the eGLs, the observed Gls, and the area under a receiver operating characteristic curve (AUROC) were used to calculate the confidence interval and confirm the model's discrimination. Then, the IAUC, GI, GL, and eGL values measured from the subjects were expressed as means and standard deviations (SD). In addition, the Pearson correlation analysis was used to see whether the observed Gls are correlated with the eGLs. In addition, the simple linear regression analysis was used to numerically confirm the effect of various variables on the GL-eGL correlation. All collected data were analyzed using IBM SPSS Statistics version 25, and the findings were tested for significance at a significance level of *p* < 0.05.

### Results

#### General Characteristics of the Subjects

**Figure 1:**
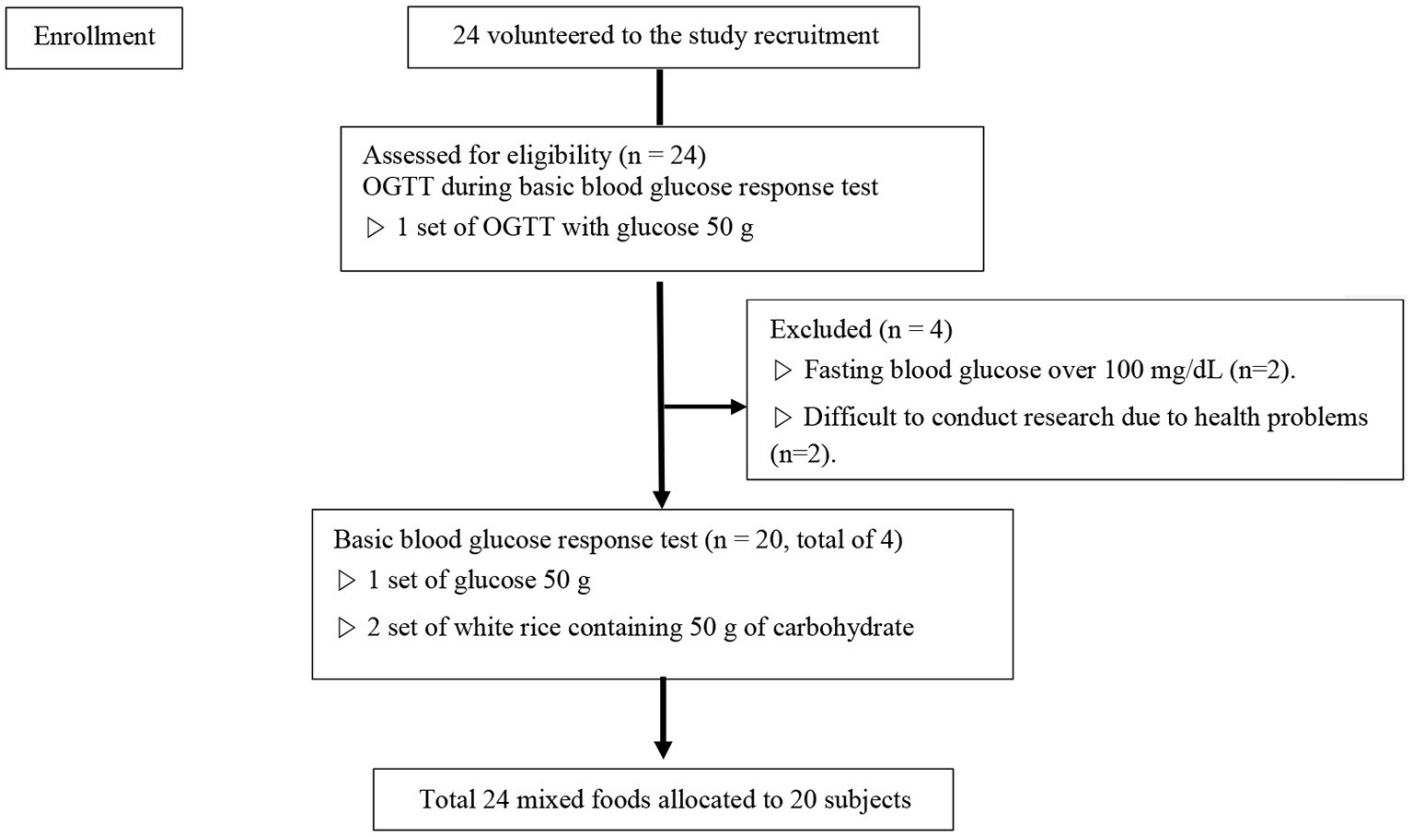
Flow chart of study process.

For this study, 24 subjects were recruited that fit the selection criteria through an open call process. Four of them were excluded during the first using through an OGTT, and the research was conducted on the remaining 20 subjects (Figure 1). Table 1 indicates the general characteristics of the subjects and the mean nutrient contents of the foods used for the study. Each item is indicated in both the mean and the SD. The subjects consisted of 20 healthy adults (ten men, ten women). The mean BMI was at normal weight category at 21.8 ± 1.92 kg/m^2^. The waist-hip ratio of the subjects was 0.8 ± 0.0, which is within the normal level (cut-offs of waist–hip ratio for the risk for abdominal obesity is male≥0.90, female≥0.85) (23). The subject's mean fasting blood glucose (measured after 10 h of fasting or longer) was within the normal range at 92.8 ± 4.78 mg/dL. The available carbohydrate, fat, protein, and fiber in all test foods used in the study were 37.9 ± 17.65 g, 10.2 ± 8.14 g, 8.5 ± 5.81 g, and 2.8 ± 2.86 g, respectively.

**Table 1:** Comparison of characteristics between validation subjects and development subjects for eGL prediction model.

	**Current validation study**	**Previously developed eGL prediction model^**c**^**	***P-*value**
**Characteristics of subjects**			
*N*	20	34	
Data collection time	July to September, 2019	April to August, 2017	
Men (%)	50	50	0.364
Age	24.3 ± 1.98	23.2 ± 2.11	0.052
Height (cm)	169.0 ± 7.69	168.6 ± 7.27	0.841
Weight (kg)	62.7 ± 9.10	64.8 ± 11.68	0.487
BMI (kg/m^2^)^a^	21.8 ± 1.92	22.7 ± 3.44	0.289
Skeletal muscle mass (kg)	27.4 ± 6.32	26.9 ± 6.27	0.749
Percent body fat (%)	22.0 ± 7.69	21.3 ± 9.33	0.762
Waist-hip ratio	0.8 ± 0.04	0.8 ± 0.05	0.392
Basal metabolism (kcal)	1,431.9 ± 221.15	1,416.4 ± 221.95	0.805
/blood glucose (mg/dL)	92.8 ± 4.78	92.7 ±5.05	0.866
**Estimated regression coefficient**			
*N*	192	239	
Available carbohydrate^b^	37.9 ± 17.65	47.6 ± 20.32	0.000
Fat	10.2 ± 8.14	9.4 ± 6.27	0.249
Protein	8.5 ± 5.81	11.6 ± 6.47	0.000
Fiber	2.8 ± 2.86	4.6 ± 3.34	0.000

*Characteristics of subjects represent M ± SD*.

a *Body Mass Index*.

b *Total carbohydrate-dietary fiber*.

c *Lee (22)*.

#### eGL Prediction Model Transportability

Table 1 shows the transportability assessment results of the eGL prediction model. There was no difference in terms of height (cm), weight (kg), BMI (kg/m^2^), skeletal muscle mass (kg), or percent body fat (%) between the subjects for validation and the subjects at the time of the model's development. On the other hand, the 24 foods selected for this validation study had significantly different nutrient contents from the 32 foods selected for the model development, except for fat (10.2 vs. 9.4 g, *p* = 0.000). Specifically, the foods used for this validation study had less protein than the foods used for model development (8.5 vs. 11.6 g, *p* = 0.000), less fiber (2.8 vs. 4.6 g, *p* = 0.000), and less carbohydrate (37.9 vs. 47.6 g, *p* = 0.000). The logistic regression analysis confirmed that the regression coefficient included in the eGL prediction model matched the coefficient at the time of the model's development (Table 1).

#### Calibration and Discrimination Assessments for the eGL Prediction Model

The Hosmer-Lemeshow goodness-of-fit test for calibration assessment and the AUROC test for discrimination assessment resulted in the findings listed in Table 2. Across all subjects, the *x*^2^ value was 11.607 and the *p*-value was 0.160. Between genders, the men reported an *x*^2^ value of 7.655, and a *p*-value of 0.468, whereas the *x*^2^ value was 9.427 and the *p*-value was 0.308 for the women. As for BMI, the *x*^2^ value was 16.498 and the *p*-value was 0.036 in people with normal BMIs (18.5–23 kg/m^2^), whereas the same values were 7.571 and 0.476 among overweight and obese people (BMI exceeding 23 kg/m^2^). As for percent body fat (%), the *x*^2^ value was 11.608 and the *p*-value was 0.170 among subjects with standard body fat, and the same values were 1.088 and 0.998 among people with higher than standard body fat (Table 2).

**Table 2:** Hosmer-Lemeshow goodness of fit test and ROC curve for eGL prediction model.

		**H-L test**		**C-statistic (95% CI)**
		***x* ^2^**	***P***	
Overall		11.607	0.160	0.571 (0.400–0.741)
Gender	Men	7.655	0.468	0.521 (0.052–0.991)
	Woman	9.427	0.308	0.589 (0.420–0.757)
BMI (kg/m^2^)^a^	≤23 (normal)	16.498	0.036	0.564 (0.400–0.727)
	>23 (overweight,	7.571	0.476	0.564 (0.068–1.000)
	obesity)			
Percent body	Average	11.608	0.170	0.543 (0.362–0.724)
fat (%)	above average	1.088	0.998	0.783 (0.672–0.894)

a *Body Mass Index*.

The discrimination of the model was assessed using the observed GL values, the eGL values from the prediction model, and the AUROC, at a confidence interval of 95%. The AUROC for all subjects was 0.571 (95% CI = 0.400–0.741), and 0.521 for men (95% CI = 0.052–0.991) and 0.589 for women (95% CI = 0.420–0.757). As for BMI, the AUROC was 0.564 (95% CI = 0.400–0.727) in the 23 kg/m^2^ or lower group (normal weight), which was the same for subjects with BMIs exceeding 23 kg/m^2^ (overweight). As for percent body fat, the AUROC was 0.543 (95% CI = 0.362–0.724) and 0.783 (95% CI = 0.672–0.894), respectively. The AUROC was the highest among participants with standard or higher percent body fat, and lowest among male subjects at 0.521 (Table 2).

#### Correlation Between GL and eGL

**Figure 2:**
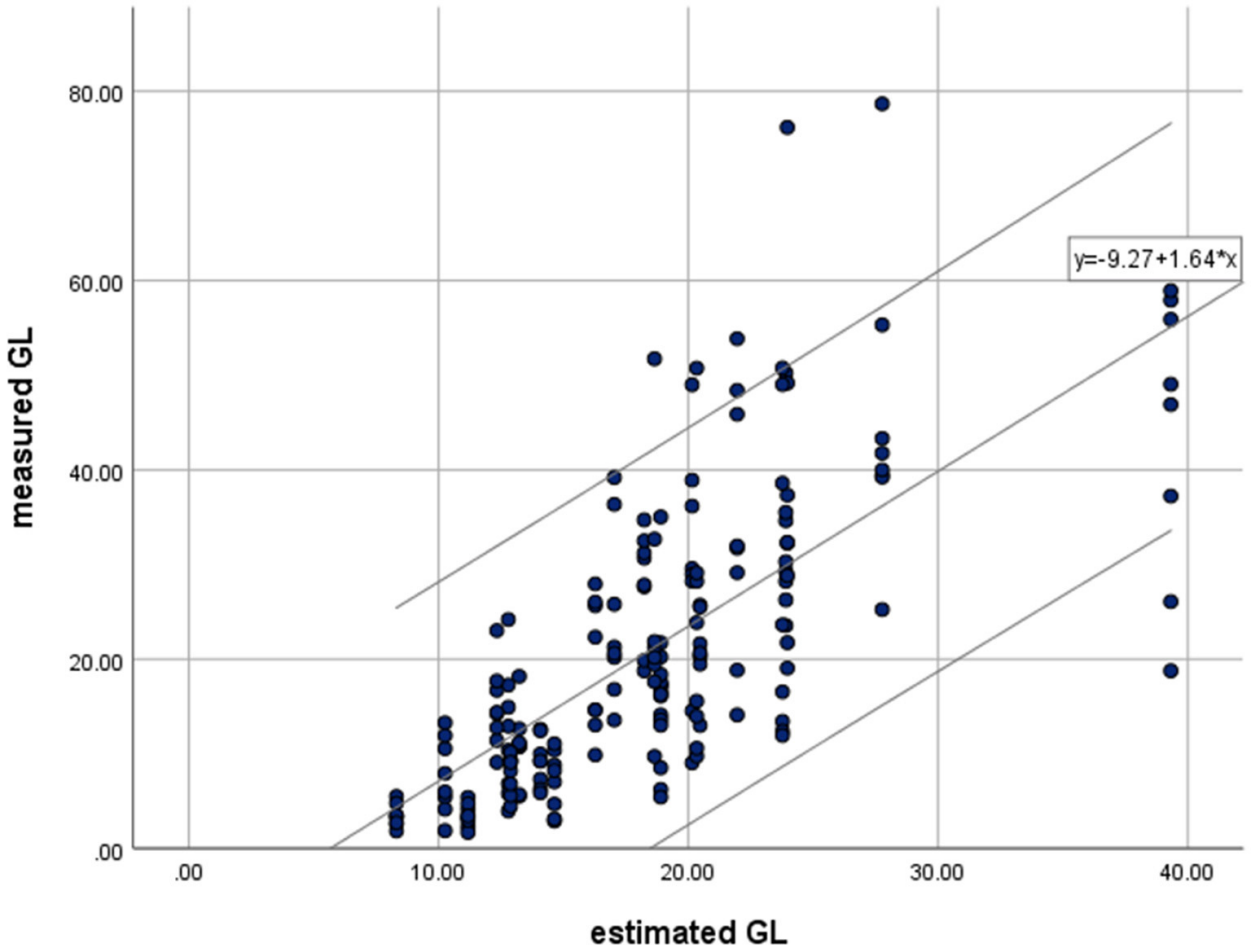
Relationships between means of measured glycemic load (GL) and estimated glycemic load (eGL) for available processed food by simple linear regression. Regression were made for all test meals (• and - : *R*^2^ = 0.507, *P* = 0.000). Values of parameter estimation.

Table 3 summarizes the findings on the GL-eGL correlation of the 24 fast foods. The correlation coefficient was statistically significant at 0.712 (*p* < 0.01), indicating a strong positive correlation. Figure 2 indicates the simple regression analysis results for GL and eGL of the 24 processed foods. According to the simple regression model, *measure GL* = −9.27+1.64 × *estimated GL*measured GL. The findings were significant at a *p* < 0.001 significance level. The eGL was found to explain 50.7% of the measured GL. In other words, when eGL increases by 1, actual GL increases by 1.637 (Table 3).

**Table 3:** Relationships between means of GL and eGL for available processed food.

		**GL**	**eGL**
GL^a^	Pearson's correlation	1	0.712^**^
	*P*-value	–	0.000
eGL^b^	Pearson's correlation	0.712^**^	1
	*P*-value	0.000	–

** *Values are significant in both sides (P < 0.01)*.

a *Glycemic index*.

b *Glycemic load*.

#### GI and GL by Food

Table 4 lists the IAUC, GI, GL, and eGL calculated based on the dietary intake and nutrient contents of the processed foods used in the study and the blood glucose measurements. Among the 24 food products, 15 products were low-GI foods (GI ≤ 55), three products were high-GI foods (GI ≥ 70), and the other six products were medium-GI foods (55 < GI < 70). The low-GI foods were: two bread products (bulgogi croquette and sponge cake); one hot dog product (cheese and sausage hot dog); three noodle products (cream pasta, spicy noodle, and tomato pasta); one salad product (corn salad); one shake product (balance shake); one soup product (button mushroom soup); and one tteokbokki product (wheat noodle tteokbokki). Bibimbap (frozen), fried rice with shrimp (frozen), and cereals were high-GI foods. Among the 24 food products, six products were low-GL foods (GL ≤ 10), 12 products were high-GL foods (GL ≥ 20), and the other six products were medium-GL foods (10 < GL < 20). Button mushroom soup, corn salad, spicy noodle, balance shake, cheese and sausage hot dog, and sponge cake were low-GL foods (Table 4).

**Table 4:** Nutrient values, IAUC, GI, GL, and eGL values of fast foods used in this study.

**Category**	**Food name (kcal/serving)**	**Carbohy-drate (g)**	**Dietary fiber (g)**	**Protein (g)**	**Fat (g)**	**IAUC^**a**^**	**GI^**b**^**	**GI sort**	**GL^**c**^**	**GL sort**	**eGL**
Bread	Bulgogi croquette (220 kcal/80 g)	25	2	9	10	1,843 ± 775	52 ± 29	Low	12 ± 7	Med	13
	Sponge cake (105 kcal/30 g)	18	0	2	2.8	1,636 ± 412	44 ± 11	Low	8 ± 2	Low	13
Calorie controlled	Balance shake (230 kcal/60 g)	31	9	20	5	1,304 ± 758	35 ± 18	Low	8 ± 4	Low	10
meal	Sweet potato healthy meal (Cold) (185 kcal/150 g)	34	0	4	3.8	2,767 ± 969	52 ± 23	Low	18 ± 8	Med	19
	Sweet potato healthy meal (Hot) (185 kcal/150 g)	34	0	4	3.8	2,348 ± 1,077	42 ± 17	Low	14 ± 6	Med	19
	Tofu lentil-rice meal (340 kcal/210 g)	50	0	19	8	2,503 ± 816	68 ± 28	Med	34 ± 14	High	22
Cereal	Cereal (150 kcal/40 g)	35	0	2	0	3,026 ± 976	83 ± 37	High	29 ± 13	High	20
	Whole-grain cereal (169 kcal/40 g)	30	1.9	2.9	4.7	2,514 ± 612	69 ± 25	Med	19 ± 7	Med	16
Dumpling	Dumplings with kimchi; frozen (407.5 kcal/220 g)	40	5.5	15.5	22.0	1,749 ± 713	31 ± 12	Low	11 ± 4	Med	13
	Dumplings with meat; frozen (467.5 kcal/200 g)	50	1.5	19.5	21.5	3,643 ± 1,172	58 ± 12	Med	28 ± 6	High	18
Easy cooked rice	Bibimbap; frozen (315 kcal/217 g)	58	7	6	8	4,406 ± 2,319	73 ± 36	High	37 ± 18	High	24
	Fried rice with hamburger steak; frozen (535 kcal/275 g)	69	7	14	24	3,258 ± 1,103	52 ± 13	Low	32 ± 8	High	24
	Fried rice with shrimp; frozen (375 kcal/225 g)	63	2	7	11	3,880 ± 1,299	74 ± 26	High	45 ± 16	High	28
Hot dog	Cheese and sausage hot dog (230 kcal/80 g)	28	2	6	11	1,885 ± 518	35 ± 10	Low	9 ± 3	Low	14
Noodle	Cream pasta (560 kcal/331.2 g)	58	2	16	30	1,429 ± 413	37 ± 7	Low	21 ± 4	High	20
	Spicy noodle (135 kcal/186.5 g)	25	2	1	3.7	1,127 ± 550	31 ± 14	Low	7 ± 3	Low	15
	Tomato pasta (290 kcal/270 g)	53	4	10	5	1,901 ± 809	55 ± 34	Low	27 ± 17	High	24
Porridge	Beef and mushroom rice porridge (155 kcal/250 g)	26	3	7	13	2,451 ± 700	65 ± 19	Med	15 ± 4	Med	12
	Red bean porridge (205 kcal/250 g)	46	10	9	0.5	2,589 ± 1,325	68 ± 35	Med	24 ± 13	High	19
Salad	Corn salad (100 kcal/115 g)	18	3	2	4.8	1,172 ± 315	23 ± 8	Low	4 ± 1	Low	11
Snack	Almond cookies (420 kcal/80 g)	48	0	8	22	1,757 ± 939	47 ± 28	Low	23 ± 14	High	20
	Dried tofu snack (310 kcal/65 g)	36	0	6	16	2,445 ± 554	67 ± 25	Med	24 ± 9	High	17
Soup	Button mushroom soup (165 kcal/190 g)	13	2	4	11	1,135 ± 194	31 ± 11	Low	3 ± 1	Low	8
Tteokbokki	Wheat noodle tteokbokki (430 kcal/140 g)	91	3.3	11.1	3	2,716 ± 1,134	50 ± 17	Low	44 ± 15	High	39

*Values of IAUC, GI, GL represent M ± SD*.

a *Incremental area under the blood glucose response curve*.

b *Glycemic index*.

c *Glycemic load*.

*In the GI sort, low GI foods (GI ≤ 55) were shown as “Low,” moderate GI foods (55 < GI < 70) as “Med,” and high GI foods (GI ≥ 70) as “High”*.

*In the GL sor, low GL foods (GL ≤ 10) were represented as “Low,” moderate GL foods (10 < GL < 20) as “Med,” and high GL foods (GL ≥ 20) as “High”*.

Table 5 summarizes the classifications based on GI and GL measurements from the foods selected for this study. Corn salad, button mushroom soup, spicy noodle, balance shake, cheese and sausage hot dog, and sponge cake were classified as low-GI and low-GI foods. Beef and mushroom rice porridge and whole-grain cereal were classified as medium-GI and medium-GL products. Bibimbap (frozen), fried rice with shrimp (frozen), and cereals were high-GI and high GL foods. Low-GI and high-GL foods included cream pasta, almond cookies, tomato pasta, fried rice with hamburger steak (frozen), and wheat noodle tteokbokki. Medium-GI and high-GL foods were dried tofu snack, red bean porridge, dumplings with meat (frozen), and tofu and lentil rice meal.

**Table 5:** Classification between measured GI and GL for one serving of provided food.

		**GI classification**		
		**Low** **(GI ≤55)**	**Medium** **(55 < GI < 70)**	**High** **(GI ≥70)**
GL classification	Low (GL ≤ 10)	Button mushroom soup Corn salad Spicy noodle Balance shake Sponge cake Cheese and sausage hot dog	–	–
	Medium (10 < GI < 20)	Dumplings with kimchi; frozen Bulgogi croquette Sweet potato healthy meal (Hot) Sweet potato healthy meal (Cold)	Beef and mushroom rice porridge Whole-grain cereal	–
	High (GI ≥ 20)	Cream pasta Almond cookies Tomato pasta Fried rice with hamburger steak; frozen Wheat noodle tteokbokki	Dried tofu snack Red bean porridge Dumplings with meat; frozen Tofu lentil-rice meal	Cereal Bibimbap; frozen Fried rice with shrimp; frozen

### Discussion

This study was conducted to validate the eGL prediction model developed in a previous study (12) by commercially available fast foods with a more diverse nutrient content, assessing the model's prediction model and performance, and using correlation analysis. The transportability assessment showed that the subjects at the time of model development had different characteristics from those of this validation study. Therefore, the model can be described as transportable. As for the performance assessment of the eGL model across all subjects, the calibration assessment found the good fit of the model. The discrimination of the prediction model was assessed at 0.571 (95% CI: 0.400–0.741). Although it is not highly accurate, as the value exceeds 0.5, the finding indicates the possibility of validating the performance and stability of the prediction model. The correlation analysis between the observed GL and the eGL across the 24 fast foods used in this study found a correlation coefficient of 0.712 and statistical significance at a significance level of 0.01, which indicates a strong positive correlation. The finding suggests that it is appropriate to use the eGL prediction model to predict GL.

The transportability of the prediction model was analyzed based on the research data at the time of the model's development. As widely recommended for transportability assessment, the characteristics of the validation subjects and those of the development subjects were directly compared (21). The body measurement items of the validation subjects and the development subjects were not significantly different, which can be attributed to the fact that healthy subjects with less blood glucose response fluctuations were selected for both studies for higher accuracy, as typically recommended for blood glucose studies (16). However, the available carbohydrate, protein, and fiber content were different between the development study and this validation study, which indicates the generalizability of the developed model. Therefore, the model can be described as transportable.

The *x*^2^ values from the Hosmer-Lemeshow test indicate the goodness-of-fit of the model, which shows the congruence between the actually observed dependent variables and the predictions from the model (18). An *x*^2^ value close to 0 indicates a higher level of goodness-of-fit. The model is statistically significant if the significance probability is higher than a significance level of 0.05(24, 25). Across all subjects, the *x*^2^ value was 11.607 and the *p*-value was 0.160, indicating that the prediction model is a good fit with the observed values. Between genders, the men reported an *x*^2^ value of 7.655, and a *p*-value of 0.468, whereas the *x*^2^ value was 9.427 and the *p*-value was 0.308 for the women. The observed GLs were congruent to the eGLs under both classifications, however, men group had higher level of goodness-of-fit than women. As for BMI, the *x*^2^ value was 16.498 and the *p*-value was 0.036 in people with normal BMIs (18.5–23 kg/m^2^), which means the prediction model was not statistically significant. The same values were 7.571 and 0.476 among overweight and obese people (BMI exceeding 23 kg/m^2^), indicating that the model is a good fit. As for percentage of body fat, the prediction models were found to be statistically significant in both groups. However, Percent body fat above average group had higher goodness-of-fit in the prediction models than percent body fat average group according to *x*^2^.

It has been suggested that an AUROC of “0.5 or higher and below 0.7” indicates low accuracy, “0.7 or higher and below 0.9” indicates medium accuracy, and “0.9 or higher and below 1.0” indicates high accuracy. A higher AUROC value indicates a higher level of discrimination (21, 26). The AUROC for all subjects was 0.571 (95% CI = 0.400–0.741), which indicates low discrimination accuracy at 57.1%. The discrimination accuracy was similar between the two genders: 52.1% for men and 58.9% for women. As for the classifications based on BMI, in both below 23 or above 23, discrimination accuracy was at 56.4%. As for percent body fat, the group with standard body fat reported a discrimination accuracy of 54.3%, whereas it was 78.3% for the subjects with higher-than-standard body fat. The prediction model was found to be less accurate across all subjects, and consistent findings were observed across genders, BMI groups, and body fat groups. The AUROC assessment found a discrimination accuracy exceeding 0.5 across all classifications, which confirms that the performance and stability of the prediction model can be discriminated across all classifications.

The correlation coefficient between GLs and eGLs measured from the 24 processed foods was statistically significant at 0.712 (*P* < 0.01), indicating a strong positive linear relationship. Kim et al. (27) applied foods' nutrient contents and GL measurements to the GL to compare GL and eGL values. They found a correlation coefficient of 0.866 indicating a strong and statistically significant (*p* < 0.01) positive correlation. The findings suggest that GL measurements can be predicted by applying the nutrient contents from other previous studies to the eGL model.

Assuming that the classifications for eGL are the same as the GL classifications, balance shake and button mushroom soup were classified as low-GL foods. They were also classified as low-GL foods in the estimated GL prediction model. Bulgogi croquette, sweet potato healthy meal (cold/hot), whole-grain cereal, dumplings with kimchi (frozen), and beef and mushroom rice porridge were found to have medium GL. These foods were also classified as medium-GL foods in the estimated GL prediction model. Tofu and lentil rice meal, cereal, bibimbap (frozen), fried rice with hamburger steak (frozen), fried rice with shrimp (frozen) cream pasta, tomato pasta, almond cookies, and wheat noodle tteokbokki were found to have high GL. These foods were also classified as high-GL foods in the estimated GL prediction model. Sweet potato healthy meal (cold/hot), dried tofu snack, and cereals were found to have different GIs depending on their protein, fat, and fiber content, despite the fact that their carbohydrate contents are similar. These findings are similar to those reported by Sun et al. (28), who reported that consuming white rice, oil 30 g, chicken protein 20 g, and vegetable 120 g results in lower blood glucose response and GI than consuming white rice (with available carbohydrate of 50 g). The findings are also similar to those reported by Quek et al. (29), who reported a significant decline in blood glucose response when consuming white rice with tofu (bean protein).

High-GI and high-GL foods are digested and absorbed faster and create faster blood glucose response, resulting in a rapid increase in early blood glucose levels (30). Therefore, caution is advised when consuming these foods. In addition, the GI ≤ 55 and 55 < GI < 70 sections and the GL ≤ 10 and 10 < GL < 20 sections indicate low-GI foods, low-GL foods (3), and medium-GL/medium-GI foods, which are characterized by slower digestion and absorption and slower blood glucose response, and can be safely consumed.

The findings of this study confirmed a strong correlation between eGL values and GL measurements based on available carbohydrate, protein, fat, and fiber. This suggests that GL values can be predicted using the eGL prediction model and food nutrient contents, instead of repeatedly taking blood glucose measurements. As such, the model is expected to contribute to facilitating GL measurement. The model is expected to be particularly helpful for Koreans by providing quantitative and qualitative information on carbohydrate intake. Our model provides accurate information on the GLs of the foods recently preferred by Koreans, who tend to eat out more and consume more fast foods. The information will be useful for patients and people requiring weight control, and contribute to prevention and management of chronic diseases.

As for the limitation of this study, first, although using finger-prick glucose test is a well-established procedure, used widely in hospitals as a standard practice, there is still a possibility of low accuracy by measuring blood sugar by self-blood glucose meter. Second, the subject criteria for the validity verification study and the subject criteria at the time for the development of the prediction model were similar, which is not the best choice for an external feasibility study.

To our knowledge this is the first study not only in Korea, but, also globally that suggest a simplified blood glucose prediction model. This equation, especially, may serve as a convenient blood glucose management method for diabetic patients, people with impaired glucose tolerance, and people seeking prevention and management of chronic diseases. We can assure that this model facilitates GL prediction, and promotes understanding of blood glucose control among people in need of, or interested in, blood glucose control, and helps people with their food choice and health management in general.

### Conclusion

With the increase of consumers purchasing fast foods compared to home-cooked meals, the findings of this study suggest that this prediction model will greatly contribute to healthy food choices because it allows customers to predict blood glucose responses based on the readily available nutrient label.

## Data Availability Statement

The raw data supporting the conclusions of this article will be made available by the authors, without undue reservation.

## Ethics Statement

The studies involving human participants were reviewed and approved by KyungHee University, Global Campus IRB. The patients/participants provided their written informed consent to participate in this study.

## Author Contributions

YP, S-JC, and KN conceptualized and designed the study. ML, KN, and S-JC selected the test foods and conducted the statistical analyses. ML and YP recruited participants, performed the experiment, and prepared the original draft. HK Interpretation of the data and prepared the manuscript in English. ML, HK, and YP finalized the manuscript. All authors listed have made substantial, direct, intellectual contribution to the work and approved it for publication.

## Funding

This research was partially funded by Pulmuone Inc, and BK21 plus program, AgeTech-service convergence major through the National Research Foundation (NRF) funded by the Ministry of Education of Korea (5120200313836).

## Conflict of Interest

KN was employed by Pulmuone Inc. The remaining authors declare that the research was conducted in the absence of any commercial or financial relationships that could be construed as a potential conflict of interest. The funder was not involved in collection, analysis, interpretation, or decision to any of the process for publication.

## Publisher's Note

All claims expressed in this article are solely those of the authors and do not necessarily represent those of their affiliated organizations, or those of the publisher, the editors and the reviewers. Any product that may be evaluated in this article, or claim that may be made by its manufacturer, is not guaranteed or endorsed by the publisher.
